# The BDNF–TrkB–CREB Signalling Pathway Is Involved in Bisphenol S-Induced Neurotoxicity in Male Mice by Regulating Methylation

**DOI:** 10.3390/toxics10080413

**Published:** 2022-07-23

**Authors:** Yi-Zhou Li, Zi-Yao Wu, Bi-Qi Zhu, Yu-Xiao Wang, Ya-Qi Kan, Huai-Cai Zeng

**Affiliations:** 1 Guangxi Key Laboratory of Environmental Exposomics and Entire Lifecycle Health, Guilin Medical University, Guilin 541199, China; 18300926685@163.com (Y.-Z.L.); ziyaowu@163.com (Z.-Y.W.); wangyuxiao0629@163.com (Y.-X.W.); kyq8426@163.com (Y.-Q.K.); 2 Guangxi Health Commission Key Laboratory of Entire Lifecycle Health and Care, Guilin Medical University, Guilin 541199, China; 3 Department of Environmental and Occupational Health, School of Public Health, Guilin Medical University, Guilin 541199, China; 4 Department of Preventive Medicine, School of Public Health, University of South China, Hengyang 421001, China; fucunjidao@163.com

**Keywords:** bisphenol S, neurotoxicity, methylation regulation, BDNF–TrkB–CREB signalling pathway

## Abstract

Bisphenol S (BPS), the most common substitute for bisphenol A in manufacturing, is associated with neurotoxicity, but its molecular mechanisms are unclear. Here, we studied the role of the BDNF–TrkB–CREB (brain-derived neurotrophic factor–tropomyosin-related kinase B–CAMP response element-binding protein) signalling pathway in bisphenol S-induced neurotoxicity via methylation regulation in male C57BL/6 mice. The mice were treated with sesame oil or 2, 20 and 200 mg/kg body weight BPS for 28 consecutive days, and the hippocampus was extracted. We recorded the body weight, organ index, and hippocampal pathology and ultrastructure of the mice. The BDNF, TrkB, CREB, phosphorylated (p)-CREB, DNMTs (DNA methyltransferases) levels were determined by qRT-PCR and/or Western blotting. BDNF promoter IV methylation level was detected by bisulfite sequencing PCR. BPS damaged the mouse hippocampus ultrastructure and reduced the number of synapses. Further, it increased the methylation rate of BDNF promoter IV; downregulated BDNF, CREB, p-CREB/CREB and DNMT1 expression; and upregulated DNMT3a and DNMT3b expression. Therefore, we speculate that the BDNF–TrkB–CREB pathway may be involved in BPS-induced neurotoxicity in male mice by regulating methylation.

## 1. Introduction

In humans, BPA exposure can lead to diabetes, asthma, cancer, reproductive toxicity, neurotoxicity, hepatotoxicity and other toxic effects [1,2]. Due to these harmful effects, in 2011, the European Commission restricted the use of BPA in infant feeding bottles [3]. Bisphenol S (BPS) is a substitute of industrial bisphenol A (BPA), a typical exogenous endocrine disruptor and an important material for producing epoxy resin, polycarbonate and synthetic resins. Due to the widespread use of BPS, it may cause a greater environmental and physical burden, and damage to human health. It is reported that the concentration of BPS in the water of the Yangtze River in China reached 14.9 ng/L, and the concentration in the blood of residents reached 169 ng/mL [4]. The levels of BPS in human blood and urine are very close to the levels of BPA [5]. Compared with BPA, BPS has a higher absorption rate, a longer biological half-life [6], similar endocrine activity and placental damage ability to BPA, and can be released from the baby bottle [7]. Therefore, BPS may not be a safe substitute for human health [8]. 

In recent years, there have been many studies on the harmful effects of BPS. BPS can increase the triglyceride and thyroid hormone levels in rat blood and thus increase the risk of obesity [9]. In mice, sub-chronic exposure to BPS can induce liver injury and oxidative stress [10]. Exposure to low levels of BPS can affect the feedback regulatory circuits of the hypothalamic–pituitary–gonadal axis and impair the development of zebrafish offspring [11]. In male mice, postnatal exposure to 50 µg/kg or 10 mg/kg BPS has harmful implications for reproductive function, including low sperm concentration, motility and steroid hormone levels [12]. In addition, many studies have shown that BPS could damage nerve cells in vitro [13,14], and change animal behaviour and induce neurotoxicity [15]. For example, prenatal exposure to BPS reduced fertility and changed maternal behaviour in female rats [12,15,16]. Epidemiological survey indicated that prenatal exposure to BPA and BPS may affect child neurodevelopment [17]. Salahinejad et al. found that chronic exposure to BPS affected cognitive behaviours in adult female zebrafish, with downregulation in the expression and activity of relevant genes and proteins involved in the glutamatergic–ERK–CREB (CAMP response element-binding protein) signalling cascade [18]. BPS affects the expression of 5α-reductase and the dopamine–serotonin system in the prefrontal cortex of infant female rats [19]. It also causes anxiogenic effects in mice after 10-week exposure via drinking water [20]. As the main substitute of BPA, the safety of BPS is suspect. Moreover, the other potential effects of BPS on the nervous system and its mechanism are still unclear.

Brain-derived neurotrophic factor (BDNF) is mainly expressed in the nervous system, and aids neuron development, survival and differentiation during the development of the central nervous system. It also plays an important role in mature neurons, synaptic plasticity and learning and memory in humans by binding with its specific receptor tropomyosin-related kinase B (TrkB) [21]. CREB is an important regulator of BDNF-induced gene expression in embryonic cortical neurons, and is involved in regulating neuronal growth and development, synaptic plasticity and long-term memory formation. CREB is activated by phosphorylation at serine 133 to inhibit apoptosis and to promote cell differentiation and repair after injury. CREB is phosphorylated after BDNF–TrkB signalling has been activated and therefore upregulates the expression of the BDNF gene [22].

DNA methylation is an epigenetic regulation that regulates genes without altering their sequences and mainly occurs on the cytosine of the 5′CpG3′ dinucleotide sequence. DNA methyltransferase (DNMTs), including DNMT1, DNMT2 and DNMT3 (DNMT3a, DNMT3b), plays a vital role in DNA methylation. The majority of cancer genes and/or tumour suppressor genes undergo extensive demethylation and/or methylation, which leads to tumour development. Some environmental pollutants, such as diethylstilbestrol, can change the genetic imprint of genes by changing the DNA methylation patter, thereby greatly increasing the risk of uterine cancer in female offspring [23]. Bisphenol compounds also cause epigenetic changes. For example, perinatal and lactation exposure to BPA decreased BDNF gene expression and increased the probability of DNA methylation in the hypothalamus and hippocampus of adult rats [24]. BPS can also change the methylation state of the CDH1, SFN and TNFRSF10C promoters in MCF-7 cells, which indicates that the epigenetic change induced by BPS may play a role in the development of mammary cancer [25].

Based on this evidence, and with the aim of improving understanding of the underlying molecular mechanisms of BPS neurotoxicity, we conducted this study to explore the neurotoxicity of BPS on adult C57BL/6 mice and the effect of epigenetic regulation involving the BDNF–TrkB–CREB signalling pathway on BPS-induced neurotoxicity.

## 2. Materials and Methods

### 2.1. Chemicals

BPS (99% purity) was purchased from Sigma-Aldrich (Shanghai, China). Sesame oil (Jinlongyu, Shenzhen, China) was stored in glass bottles without bisphenol compounds. Phenylmethylsulfonyl fluoride (PMSF), 4% paraformaldehyde, 2.5% glutaraldehyde, BCA kit and anti-BDNF (Cat No: GB11559) antibody were purchased from Servicebio Technology (Wuhan, China). Anti-TrkB (Cat No: 13129-1-AP), anti-β-actin (Cat No: 20536-1-AP) and anti-CREB (Cat No: 12208-1-AP) antibodies were purchased from Proteintech (Chicago, IL, USA). Anti–phosphorylated (p)-CREB (Cat No: ab32096), anti-DNMT3a (Cat No: ab188470) and anti-DNMT3b (Cat No: ab2851) antibodies were purchased from Abcam (Shanghai, China). Anti-DNMT1 (Cat No: 5032) antibody was purchased from CST (Wuhan, China). The tissue genomic DNA extraction kit (Cat No: DP304) was purchased from Tiangen Biotech (Beijing, China). The other chemicals used in this study are commercially available at the required grade.

### 2.2. Animals and Treatment

**Figure 1:**
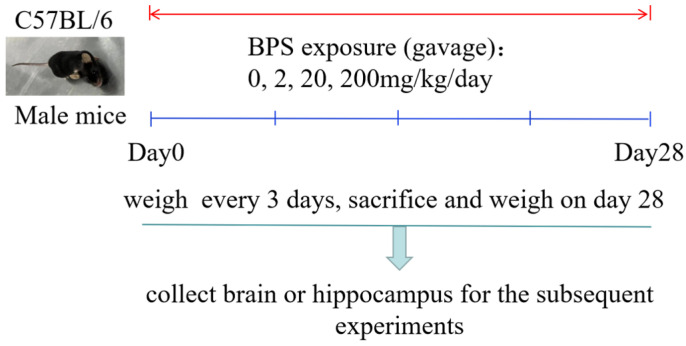
Schematic diagram of the experimental model.

C57BL/6 male mice (body weight (BW), 20–22 g, age, 8 weeks) were purchased from Hunan SJA Laboratory Animal Co., Ltd. (Changsha, China) and maintained in cages under a reversed light:dark 12:12 cycle with a set temperature of 22–25 °C and 50–60% humidity, with free access to food and water. After 1 week of acclimatization, the mice were randomly divided into four groups (mice per group, n = 10). The mice were orally exposed (gavage, 0.1 mL/10 g BW) to BPS dissolved in sesame oil (2, 20 or 200 mg/kg BW) or only sesame oil as a vehicle control at 10:00 am daily for 28 consecutive days and were weighed every 3 days. The mice were killed by cervical vertebra dislocation on day 28. The brain or hippocampus was collected for the subsequent experiments or stored in a −80 °C cooler until biochemical analysis (Figure 1). The experiments were approved by the Guilin Medical University experimental animal ethics committee, and all possible efforts were made to minimize the number of animals used and their suffering. 

### 2.3. Haematoxylin and Eosin (HE) Staining

To observe the effect of the BPS and control treatment on morphology, mouse brains that had been dissected out after the mice had been killed were weighed and fixed overnight in 4% paraformaldehyde to observe the effect of BPS on the hippocampal neurons. After fixation, brains were embedded in paraffin wax and blocks were prepared for microtomy. Sections (3-µm thick) were obtained from the tissues and dehydrated in alcohol, followed by two washes in xylene for clearing. The sections were air-dried for 30 min and then transferred to a paraffin oven for complete deparaffinization. After rehydration in descending alcohol grades, the sections were stained with HE before they were observed under an optical microscope (Nikon, Tokyo, Japan).

### 2.4. Transmission Electron Microscopy (TEM)

The ultracellular structure of the neurons, synapses and myelin sheaths in the mouse hippocampus from the control and BPS groups was assessed using TEM. After the mice had been killed, the hippocampus was collected and cut into 0.2 × 0.2 × 0.2 cm^3^ pieces, and fixed in 2.5% glutaraldehyde overnight at 4 °C. The pieces were washed three times for 15 min each with phosphate buffer (pH 7.4), and then post-fixed in 1% osmium tetroxide 0.1 M phosphate buffer for 2 h under ambient conditions. The sections dehydrated in graded ethanol solutions from 40% to 95% (each for 10 min) and then 100% acetone (three times, each for 10 min). The samples were infiltrated with a mixture of 50% epoxyresin, 50% pure acetone for 30 min at room temperature. Each sample was placed on a Teflon support, covered with a capsule containing pure epoxy resin at 60 °C for 1 h and polymerized at 80 °C overnight. After the samples were trimmed, they were sectioned into slices with 70 μm, stained with uranyl acetate and lead citrate. After drying, the ultrastructural features of synapses of hippocampus were viewed using transmission electron microscopes (Hitachi, Tokyo, Japan). We randomly selected five fields of vision at ×5000 magnification to calculate the number of synapses in each group. The number of synapses were converted into synapse numbers per area (10 μm^2^).

### 2.5. Real-Time Quantitative PCR (qRT-PCR)

The expression of BDNF, TrkB, CREB, DNMT1, DNMT3a and DNMT3b at mRNA levels in mice hippocampus were analyzed by qRT-PCR. Total RNA of mice hippocampus was extracted by Trizol Regent. The purity of nucleic acid was evaluated by the optical density ratio of RNA samples at 260 nm and 280 nm, RNA quality was assessed by running on a gel, and the total RNA concentration was evaluated according to the absorbance at 260 nm. cDNA synthesis for mRNA was performed with 2 µg of total RNA by using the Thermo Scientific Revert Aid First Strand cDNA Synthesis Kit according to the manufacturer. The cDNA was stored at 80 °C until use. The sizes of products and their corresponding primer sequences were listed in Table 1. The PCR procedure included an initial denaturation at 95 °C for 10 min, followed by 40 cycles of 95 °C for 15 s (denaturation), 60 °C for 30 s (annealing), and 72 °C for 30 s (extension). A dissociation curve analysis was performed for each gene to examine the amplification of the non-targeted fragment. Only one peak was observed for each reaction, indicating that only the target gene was amplified. The 2^−^^∆∆Ct^ (Ct, cycle threshold) method was used to calculate the fold changes in relative target gene expression.

### 2.6. Western Blotting

BDNF, TrkB, CREB, p-CREB, DNMT1, DNMT3a, DNMT3b and β-actin expression were analyzed by Western blotting. Hippocampal tissue (a mouse), 1 mL RIPA lysis buffer (Servicebio Technology, Wuhan, China; Cat No: G2002), 10 μL PMSF (Servicebio Technology, Wuhan, China; Cat No: G2008) and 20 μL protease inhibitor cocktail (Servicebio Technology, Wuhan, China; Cat No: G2006) were added, and the tissue was ground thoroughly. Then, the sample was centrifuged at 4 °C 12,000 rpm for 10 min and the supernatant was removed. Total protein was quantified with a bicinchoninic acid (BCA) kit according to the instruction manual. The loading quantity of each protein was 40 μg. Sodium dodecyl sulfate–polyacrylamide gel electrophoresis was performed, and the proteins were transferred to nitrocellulose membranes. After blocking with 10% non-fat milk, the proteins were probed with primary antibodies against BDNF (1:1000), TrkB (1:1500), p-CREB (1:1500), CREB (1:1000), DNMT1 (1:1000), DNMT3a (1:1000), DNMT3b (1:2000) and β-actin (1:2000) at 4 °C overnight. HRP (1:3000) was used as the secondary antibody. Finally, the membranes were incubated for 3 minutes with Super ECL Plus (Beyotime, Shanghai, China) and developed in a gel imaging system (Tanlon, Shanghai, China). Image J software was used to analyze the gray level of the scanned image.

### 2.7. Bisulfite Sequencing PCR

BDNF promoter IV methylation level is negatively correlated with BDNF expression level and sensitive to exogenous pollutants [26,27]. In the present, the methylation rate of BDNF promoter IV in mouse hippocampus was analyzed by bisulfite sequencing PCR. The forward and reverse primers for BDNF promoter IV (product size, 439 bp) were 5′-GAAAATATTTATAAAGTATGTAATGTTTTGGA-3′ and 5′-CTATATATTTCCCCTTCTCTTCAATTAAA-3′, respectively. The hippocampal tissue was treated as a cell suspension for DNA extraction with the tissue genomic DNA extraction kit. The supernatant was aspirated, and the solution was collected in a centrifuge tube. The DNA was then treated with bisulfite and PCR-amplified. Electrophoresis (1× TAE buffer, 2.0% agarose, 5 V/cm) was performed after recovering the glue, establish the connection system and connect overnight at 16 °C, then cloned into pCRII vector using TA Cloning Kit Dual Promoter (Invitrogen, Grand Island, NY, USA). Plasmid DNA from 10 colonies were prepared using TIANprep Mini Plasmid Kit (TIANGEN, Beijing, China) and sequenced.

### 2.8. Statistical Analysis

Data are expressed as the mean ± SD. All statistical analyses were performed using SPSS for Windows (Version 28.0; SPSS Inc., Chicago, IL, USA). Statistical significance was identified by analysis of variance (ANOVA) followed by the least significant difference (LSD) test or the Dunnett T3 multiple comparisons test. *p* < 0.05 was considered statistically significant.

## 3. Results

### 3.1. The Effect of BPS on Mouse Body and Brain Weight

The mice and their brains were weighed to calculate the organ index after 28 consecutive days of BPS treatment (Table 2). There were no significant differences in the BW, brain weight and organ index between the BPS groups and the control group.

### 3.2. The Effect of BPS on Hippocampal Histopathology

**Figure 2:**
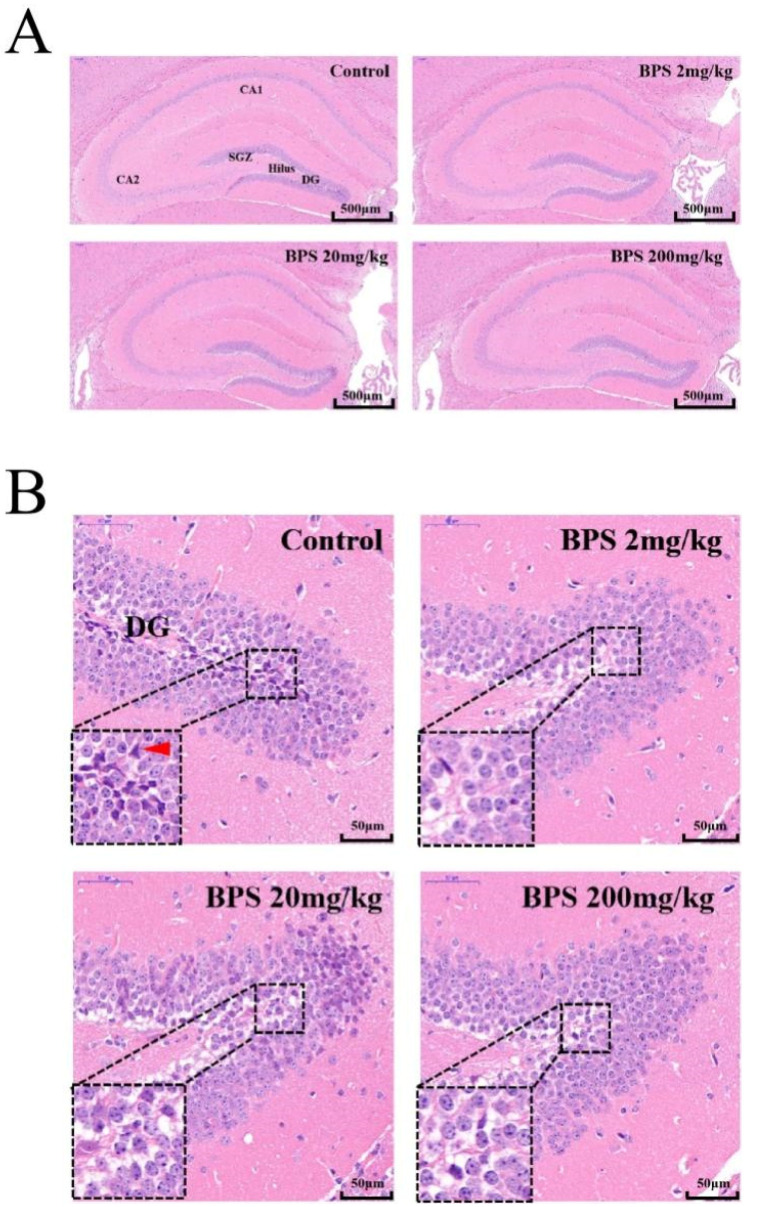
(**A**) Representative HE staining (×10) images of BPS- and control-treated mouse hippocampus. DG, dentate gyrus; CA, cornu ammonis; SGZ, sub-granular zone, Scale bar = 500 μm. (**B**) Representative HE staining (×40) images of the DG following 28-day BPS and control treatment. Red arrowhead shows pyramidal cells of the DG, Scale bar = 50 μm.

HE staining to reveal the histoarchitecture of the control and BPS mouse hippocampus showed normal morphology and structure. No obvious pathological changes were observed in the hippocampus (Figure 2A). We did not see pyramidal cells in the dentate gyrus of the hippocampus of the BPS-treated mouse, thus BPS may affect the development of the hippocampus (Figure 2B).

### 3.3. The Effect of BPS on Mouse Hippocampal Ultrastructure

**Figure 3:**
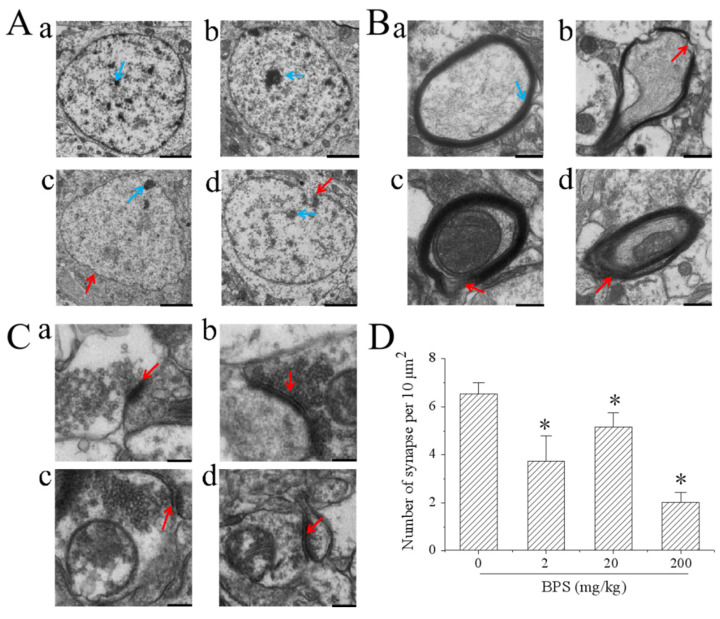
BPS caused ultrastructural changes of the nucleus, myelinated axons and synapses in mouse hippocampus after the 28-day control and BPS treatment. (**A**) Representative TEM images showing morphological nuclear changes. Red arrows show irregular nuclear envelope; blue arrows show the nucleolus; (a), control; (b), 2 mg/kg; (c), 20 mg/gk; (d), 200 mg/kg. Scale bar = 2 μm. (**B**) Representative TEM images show the ultrastructure of myelinated axons. Blue arrow shows normal myelinated axons; red arrows indicate axons with uncompacted myelin sheath; (a), control; (b), 2 mg/kg; (c), 20 mg/gk; (d), 200 mg/kg. Scale bar = 0.5 μm. (**C**) Representative TEM images show the ultrastructure of the synapses. Red arrows indicate normal synapses with construction of the presynaptic membrane, postsynaptic membrane and synaptic cleft; (a), control; (b), 2 mg/kg; (c), 20 mg/gk; (d), 200 mg/kg. Scale bar = 0.2 μm. (**D**) Effect of BPS on the number of synapses. The data are expressed as the mean ± SD (n = 5). Compared with the control group * *p* < 0.05.

We found that BPS exposure was sufficient to induce ultrastructural changes in the hippocampus region. Hippocampal neuron nuclear morphology was altered by 20 and 200 mg/kg BPS (Figure 3). After treatment with 200 mg/kg BPS, the nucleolus was not obvious compared with that of the other treatment groups (Figure 3A). In addition, BPS affected ultrastructural changes in myelin and the axons. In the BPS groups, the myelin sheath around many of the axons was incomplete when compared with that of the control group (Figure 3B). Further, BPS reduced the number of synapses without affecting the synaptic structure (Figure 3C,D). These results indicate that BPS affects neuron morphology, axon myelination and synapse formation in mouse hippocampus.

### 3.4. The Effect of the BDNF–TrkB–CREB Pathway on BPS-Induced Neurotoxicity

**Figure 4:**
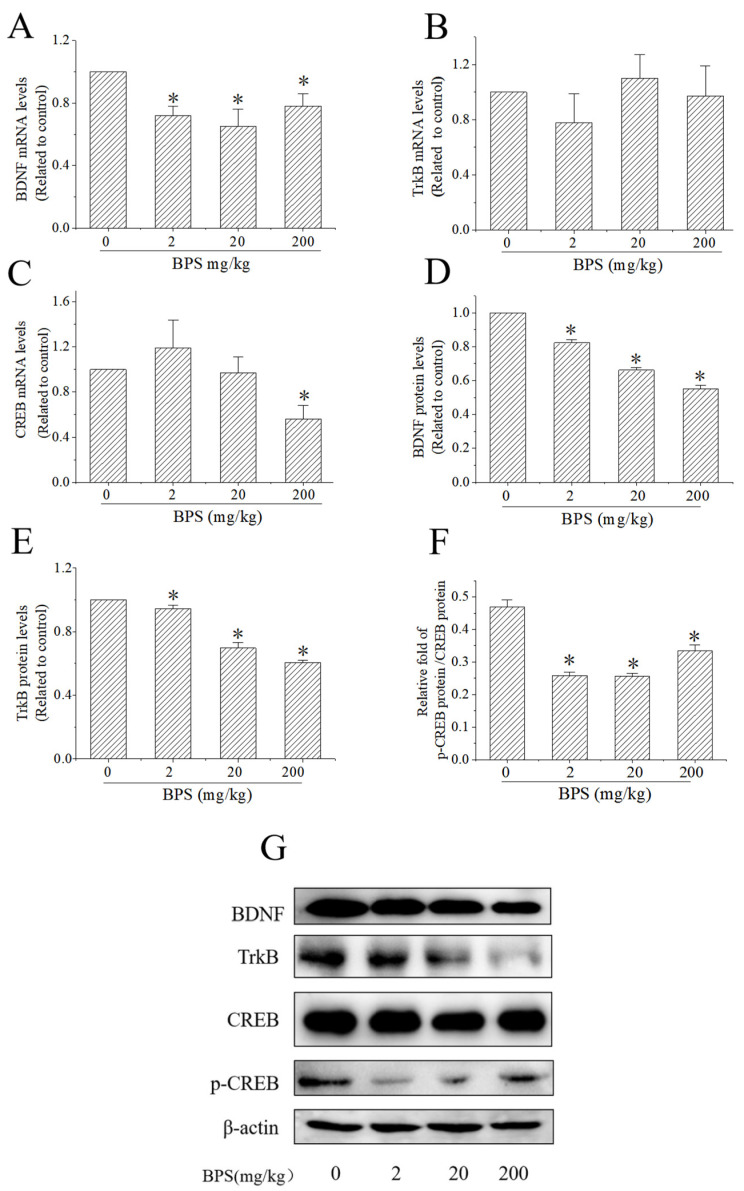
Effect of BPS on BDNF, TrkB, CREB and p-CREB expression in mouse hippocampus. (**A**) Expression of BDNF mRNA. (**B**) expression of TrkB mRNA. (**C**) Expression of CREB mRNA. (**D**) Expression of BDNF protein. (**E**) expression of TrkB protein. (**F**) Ratio of p-CREB protein/CREB protein. The data are expressed as the mean ± SD (*n* = 3); compared with the control group * *p* < 0.05. (**G**) Grayscale scan of BDNF, TrkB, CREB and p-CREB expression measured by Western blotting.

The mRNA levels of BDNF, TrkB and CREB were detected by qRT-PCR and their protein levels were detected by Western blotting. The p-CREB protein level was detected by Western blotting. When compared with the control group, the mRNA levels of BDNF decreased significantly in the 2, 20 and 200 mg/kg BPS groups (Figure 4A). There was no significant difference between the BPS and control group for the mRNA expression of TrkB (Figure 4B), and BPS decreased the CREB mRNA expression with 200 mg/kg BPS treatment (Figure 4C). Western blotting showed that the protein expression of BDNF and TrkB gradually decreased as the BPS concentrations increased (Figure 4D,E). In addition, p-CREB protein/CREB protein expression was decreased after BPS treatment (Figure 4F), indicating that BPS not only reduced the expression of CREB in mouse hippocampus, but also inhibited the activation of CREB.

### 3.5. The Effect of BPS on BDNF Promoter IV Methylation in Mouse Hippocampus

**Figure 5:**
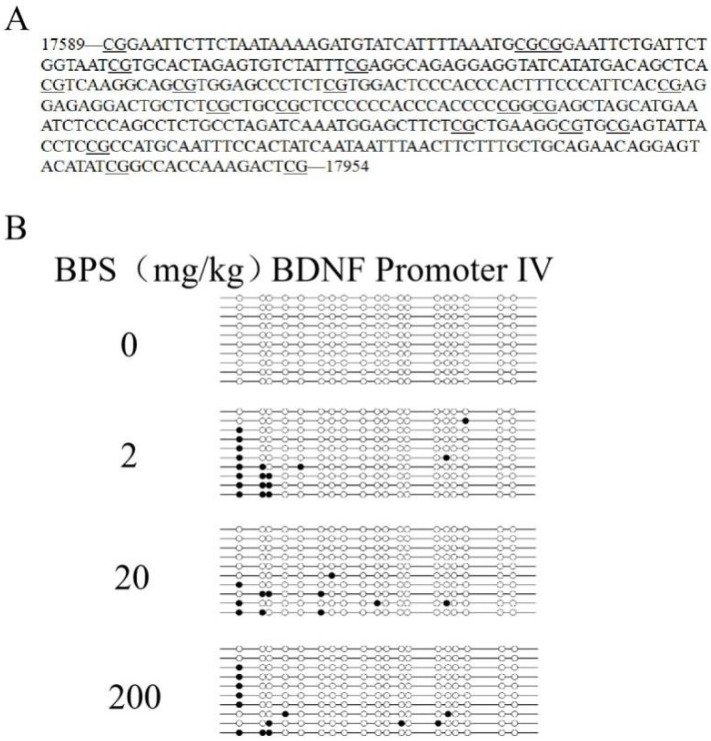
Bisulfite sequencing methylation analysis of individual CpG dinucleotides of BDNF promoter IV from mouse hippocampus. (**A**) The locations of the 19 CpG sites within promoter IV are listed, and the methylation status was assessed by bisulfite sequencing. The underlined sections represent CpG dinucleotide sites. (**B**) Methylation of promoter IV. White and black circles denote unmethylated and methylated CpG sites, respectively. Each row indicates a specific plasmid clone. Ten clones were from the same group.

To explore the role of methylation in BPS-induced neurotoxicity, we investigated the methylation rate of promoter IV of the BDNF gene via bisulfite sequencing PCR (Figure 5). The total methylation rates of the control group and the 2 mg/kg, 20 mg/kg and 200 mg/kg BPS groups were 0%, 9.5%, 5.8% and 6.8%, respectively. The result shows th BPS methylated promoter IV of the BDNF gene in mouse hippocampus. 

### 3.6. The Effects of BPS on DNMT Expression in Mouse Hippocampus

**Figure 6:**
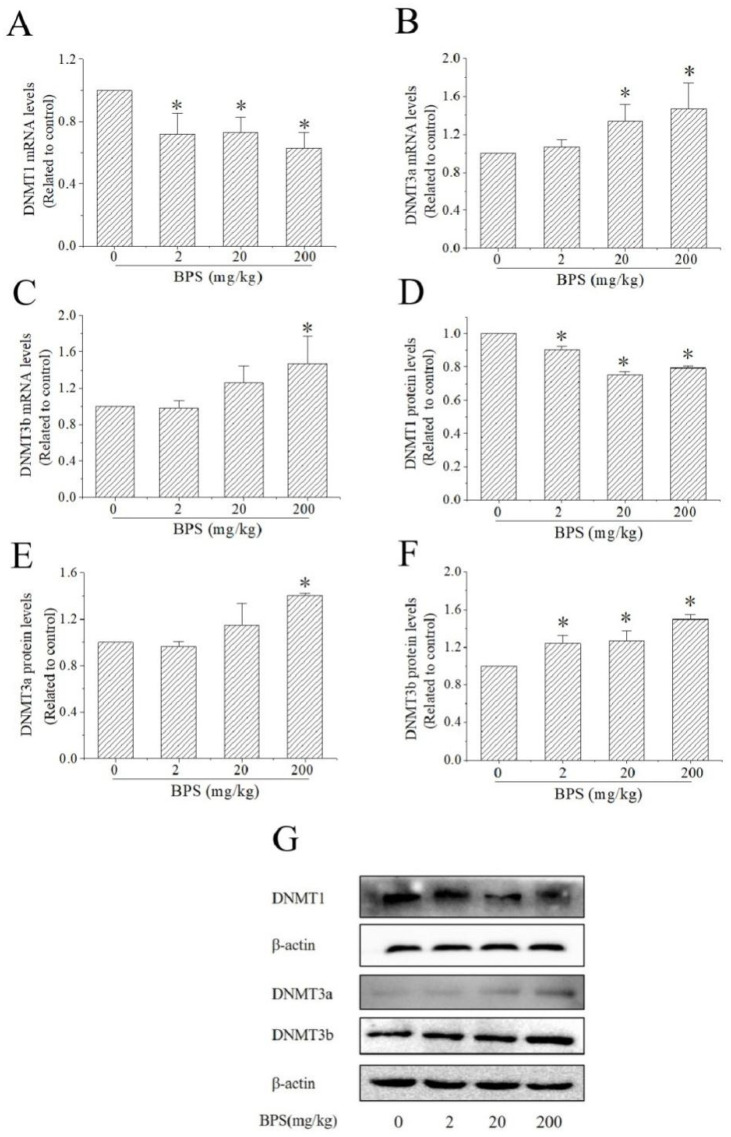
Effect of BPS on the expression of DNMTs in mouse hippocampus. (**A**) The expression of DNMT1 mRNA. (**B**) The expression of DNMT3a mRNA. (**C**) The expression of DNMT3b mRNA. (**D**) The expression of DNMT1 protein. (**E**) the expression of DNMT3a protein. (**F**) The expression of DNMT3b protein. The data are expressed as the mean ± SD (*n* = 3); compared with the control group * *p* < 0.05. (**G**) Grayscale scan of DNMT1, DNMT3a and DNMT3b expression measured by Western blotting.

As shown in Figure 6, compared with the control group, DNMT1 mRNA levels and protein levels were decreased in all BPS groups, respectively (Figure 6A,D). 20 mg/kg and 200 mg/kg BPS treatment increased the mRNA levels of DNMT3a, but only 200 mg/kg BPS treatment increased the expression of DNMT3a protein (Figure 6B,E). Compared with the control group, the mRNA levels of DNMT3b were increased in the 200 mg/kg group and BPS increased the protein levels of DNMT3b in all BPS groups (Figure 6C,F).

## 4. Discussion

BPS is one of the most important plasticizer substitutes of BPA. In recent years, the safety of BPS has been suspect. There is growing public and scientific concerns about the potential impact of BPS on neurobehavioural disorders [28]. In our study, 2, 20 and 200 mg/kg BW BPS did not significantly reduce the mouse BW and brain organ index when compared with the control treatment. HE staining showed that BPS only affected the formation of pyramidal cells in the dentate gyrus of the mouse hippocampus. However, we found that BPS induced changes in the ultrastructure of mouse hippocampus, including the decrease in synaptic counts and incompleteness of the myelin sheath. BDNF plays an important role during early and adolescent brain development and regulates learning and memory processes in the mature brain [29]. It also plays a crucial role in mature neurons, synaptic plasticity and learning and memory in humans. Therefore, we suggest that the ultrastructural changes to the mouse hippocampus may be due to the decrease in intracellular BDNF protein. Decreased BDNF mRNA and/or protein levels are related to many nervous system diseases such as Alzheimer disease, depression and autism [22].

Recent studies have shown that BPS might be toxic to the nervous system and affect animal behaviour. Embryonic exposure to low concentrations of BPS caused the damage of neural functionality to persist into adulthood [30]. BPS could also damage the mitochondrial membrane potential, lead to oxidative stress and decrease the expression of BDNF in SK-N-SH cells [14]. Oxidative stress has been involved in the debut and/or progress of several neurodegenerative disorders such as Alzheimer disease [31,32]. The BDNF–TrkB–CREB signalling pathway mediates a variety of neuronal events and plays an important role in maintaining normal brain function [33], and this signalling pathway may be involved in hippocampal neural stem cell proliferation, migration and differentiation, and affect cognitive function [34]. Liu et al. found that the BDNF–TrkB–CREB signalling pathway was involved in icariside II attenuation of cognitive impairment in Alzheimer patients [22]. Many studies have shown that the BDNF–TrkB–CREB pathway is involved in antidepressant mechanisms [35,36]. To explore the role of the BDNF–TrkB–CREB signalling pathway in BPS-induced neurotoxicity, we detected the expression of the correlated mRNA and proteins via qRT-PCR and Western blotting. We found that BDNF mRNA and protein levels decreased significantly after treatment with 2, 20 and 200 mg/kg BPS. In addition, the protein levels of TrkB and p-CREB/CREB were decreased, suggesting that BPS may reduce CREB content in the cells, leading to decreased BDNF protein and inducing neurotoxicity in mice through the BDNF–TrkB–CREB signalling pathway.

DNA methylation is an important epigenetic regulation, which has been linked to the pathogenesis of the most common neurodegenerative diseases, such as Alzheimer’s disease, Parkinson’s disease and Huntington diseases [37]. *BDNF* gene promoter IV contains the binding site for CREB, and in vitro and in vivo studies have shown that it is sensitive to DNA methylation [38]. Environmental pollutant exposures could change the BDNF promoter methylation and inhibit the expression [27,39]. Early maltreatment could produce long-lasting methylation changes in BDNF promoter IV and decrease BDNF gene expression in adult rat prefrontal cortex [26]. To explore the role of methylation in neurotoxicity caused by BPS, we studied the methylation of BDNF promoter IV in mouse hippocampus using bisulfite sequencing PCR. The results found that the methylation rate of BDNF gene promoter IV increased after BPS treatment, which may decrease the expression of BDNF. DNMTs play a critical role in learning and memory formation by regulating BDNF expression and activity. Epigenetic regulation of DNA demethylation of the BDNF gene might underlie the mechanisms of synaptic plasticity and memory retention [40]. DNMT1 is the main enzyme involved in maintaining methylation. It uses asymmetric methylated DNA as the substrate to recognize the methylated CpG sites on the parental single strand of the newly generated DNA double strand, and then catalyses the methylation of cytosine at the corresponding position of the complementary single strand to maintain DNA methylation [41]. In contrast to DNMT1, DNMT3a and DNMT3b establish DNA methylation patterns de novo [42]. The DNMT3s do not rely on DNA replication and methylate DNA bases without methylation, which is the establishment mechanism of methylation [43]. The mRNA and protein levels of DNMT1, DNMT3a and DNMT3b were detected by qRT-PCR and Western blotting. We found that the protein expression of DNMT1 was decreased and DNMT3a and DNMT3b mRNA or/and protein levels were increased, which indicated that DNMTs plays a role in BPS-induced neurotoxicity in mice and leads to abnormal methylation of the BDNF gene, finally affecting protein synthesis.

## 5. Conclusions

Our findings indicate that BPS can cause the loss of myelin sheaths and decrease the number of synapses in mouse hippocampus. In addition, BPS could decrease the expression of the BDNF gene by promoter methylation regulation. Therefore, the BDNF–TrkB–CREB pathway may mediate BPS-induced neurotoxicity in mouse hippocampus by regulating methylation. These findings provide new insight into the molecular basis of BPS-induced neurotoxicity, and lay the foundation for future studies on its molecular mechanisms.

## Figures and Tables

**Table 1:** Primer sequence of Real-Time Quantitative PCR.

Gene	Primer	Product Length (bp)
BDNF	Forward: GCCCATGAAAGAAGTAAACGTCC	136
	Reverse: AGTGTCAGCCAGTGATGTCGTC	
TrkB	Forward: AACGGAGACTACACCCTGATGG	251
	Reverse: GCAATCACCACCACGGCATA	
CREB	Forward: TGGCTAACAATGGTACGGATGG	195
	Reverse: GTGCTGTGCGGATCTGGTATGT	
DNMT 1	Forward: AATGGTGTTGTCTACCGACTGG	158
	Reverse: TTGATGTAGTCAGAATACTTGCGG	
DNMT 3a	Forward: TTGATGTAGTCAGAATACTTGCGG	154
	Reverse: AAGCCAAACACCCTTTCCAT	
DNMT 3b	Forward: CCTGCCCGCAAAGGTTTATA	101
	Reverse: AATGGACGGTTGTCGCCCT	
GAPDH	Forward: CCTCGTCCCGTAGACAAAATG	133
	Reverse: TGAGGTCAATGAAGGGGTCGT	

**Table 2:** The effect of BPS on mouse BW and brain weight.

Group	*n*	Body Weight (g)	Body Weight Gain (g)	Brain Weight (g)	Brain Index (%)
Control	10	21.83 ± 1.35	1.12 ± 0.86	0.437 ± 0.013	2.01 ± 0.09
2 mg/kg BPS	10	22.10 ± 1.14	1.16 ± 0.87	0.436 ± 0.015	1.97 ± 0.11
20 mg/kg BPS	10	22.18 ± 1.27	0.99 ± 0.66	0.443 ± 0.009	2.00 ± 0.10
200 mg/kg BPS	10	21.65 ± 1.26	1.05 ± 0.68	0.427 ±0.009	1.98 ± 0.10

## Data Availability

Data is available from the corresponding author by request.
